# Non-standard employment and mortality in Belgian workers: A census-based investigation

**DOI:** 10.5271/sjweh.3931

**Published:** 2021-03-01

**Authors:** Rebeka Balogh, Sylvie Gadeyne, Christophe Vanroelen

**Affiliations:** Interface Demography, Vrije Universiteit Brussel, Brussels, Belgium; Institute for Employment Research, University of Warwick, Coventry, UK; Health Inequalities Research Group – Employment Conditions Network (GREDS-EMCONET), Pompeu Fabra University, Barcelona, Spain

**Keywords:** accident, all-cause mortality, cancer, cause-specific mortality, fixed-term employment, precarious employment, seasonal work, suicide, temporary agency work, temporary work

## Abstract

**Objectives:**

Evidence is growing that non-standard employment is associated with adverse health. However, little is known about the relationship between different non-standard employment arrangements and subsequent all-cause and cause-specific mortality. Using population-wide data, the present study investigated this link.

**Methods:**

Data was derived from the 2001 Belgian census and a 13-year-long follow-up. The analyses comprised 1 454 033 healthy and disability-free employees aged 30–59 years at baseline. Cox regressions were fitted to analyze the mortality risks of those in non-standard employment forms (temporary agency, seasonal, fixed-term, causal work and employment program) compared to permanent employees.

**Results:**

Several groups of workers in non-standard employment arrangements in 2001 exhibited a higher mortality risk relative to permanent employees during the follow-up after adjusting for socio-economic and work-related factors. This was especially the case among men. The relative mortality disadvantage was particularly elevated for male temporary agency workers. External causes of death played an important role in this association.

**Conclusions:**

A mortality gradient between the core and outer periphery of the Belgian labor market has been observed. This study also shows that the excess risk of death, previously attributed to non-permanent employment as a whole, hides inequalities between specific forms of non-standard work (eg, temporary agency, seasonal, fixed-term employment).

The flexibilization of labor markets, weakening of the standard employment relationship, and expansion of non-standard forms of work (1–3) have led public health researchers to scrutinize whether holding any kind of job is sufficient to reap the protective health benefits ascribed to being in work (4, 5). Consequently, it has been shown that temporary employment is linked to adverse health compared to more stable jobs (6–8), although a recent study focusing on the public sector found null-effects (9). A problem, however, with using ‘temporary employment’ as a unit of analysis, is that it often serves as an umbrella concept for various contractual arrangements (10), which merit separate investigation. Moreover, it is recommendable to look again at the long-term health effects of different non-standard employment arrangements. Analogous to research on the health effects of unemployment (11), empirical support for the long-term negative consequences – or ‘scarring effects’ (12) – of job insecurity is emerging (13). The ‘scarring’ hypothesis maintains that exposure to unemployment – or, in our case, non-standard forms of employment – at one point in a career could generate effects on individuals’ health at later stages of their lives even if exposure to that labor market situation was only temporal (11, 12).

Whereas a large body of evidence has shown that the experience of unemployment spell(s) is linked to increased risk of death later on in life (14–16), only a handful of studies have focused on analyzing the relationship between temporary employment arrangements and mortality over a longer period of time (17–19). They concluded that temporary employment was linked to excess all-cause (17–19), cardiovascular and non-violent (18), as well as smoking- and alcohol-related, and external mortality (19), depending on gender, with follow-up periods of 10–16 years. However, some questions remain. First, these studies mostly compared temporary employees as a whole with permanent workers. Only in one study were involuntary and voluntary, and satisfied and unsatisfied temporary workers distinguished (17). No single study was able to distinguish between various non-permanent groups (eg, temporary agency, fixed-term workers), even though such forms of employment could have vastly different mortality implications. In general, the sample sizes of the existing studies were also limited, making cause-specific mortality analyses impossible (17), or reducing statistical power (18). One study (19) used a larger dataset, but as the sample consisted primarily of municipal employees, its findings could not be generalized to the entire employed population.

By drawing on the Belgian census and linked mortality follow-up data, we were able to eliminate some of the limitations in existing studies. Thanks to the large number of observations and a lengthy follow-up – and, as a result, a sufficient number of events occurring – we studied cause-specific mortality in a more robust way. It also follows that we were not required to group specific types of non-standard employees together in one overarching category of temporary employment. Doing so enabled us to evaluate which non-standard jobs might be more or less disadvantageous in terms of mortality (over a more than 13-year follow-up period). This can inform more targeted policy measures directed to those employment forms that are most at risk. Moreover, our strong study design, considering the ‘hard endpoint of mortality’, further added to existing evidence on adverse health effects of non-permanent employment as suggested by research using self-reported health indicators as outcomes (6, 7, 9). Belgium has had a relatively low prevalence of temporary employment over the time period investigated, in comparison to other European countries (20), providing an insightful context in which to assess the implications of non-standard employment as we could evaluate whether contractual inequalities between the sizable core and smaller periphery of salaried employees (18, 21) translate into pronounced mortality differences.

## Methods

### Data

The data for the analyses was derived from the 2001 Belgian census, linked to 13 years and three months of mortality and emigration follow-up from the National Register and death certificates between 1 October 2001 and 31 December 2014. The census covered all individuals officially registered in Belgium at the time. The causes of death were coded according to the International Statistical Classification of Diseases and Related Health Problems 10^th^ Revision.

### The study population

Individuals aged 30–59 years on the day of the 2001 census claiming to hold a job were eligible to be included in the analyses. In order to reduce the effects of health selection (5), the analysis was restricted to individuals with good or very good self-rated health and without longstanding illness or disability in 2001. To focus on the waged workers, we excluded the self-employed for whom the type of employment undertaken was systematically missing (namely ‘entrepreneurs without an employment contract’, ‘independent individuals working primarily for one person or company’, ‘those practicing another independent, liberal profession’ and those ‘helping a self-employed’), as well as any further workers who were employing workers as employers themselves. The final study population with complete information on all key variables included 1 454 033 individuals (810 981 men and 643 052 women), representing 18 828 450 person-years in total; 37 487 individuals were censored on their date of emigration.

### Measures

Our main exposure variable was derived from the following question in the census, pertaining to the individual’s main job: “In case you are a salaried employee, what kind of work do you undertake?”. The options were (i) permanent, (ii) temporary agency (*emploi d’intérimaire*), (iii) seasonal and (iv) fixed-term work, as well as (v) employment program (*programme de mise au travail*), (vi) apprenticeship/internship, (vii) student job, and (viii) casual work (without formal contract) or other. Those in permanent employment were taken as the reference category. Due to their small numbers, individuals in apprenticeships and student jobs were excluded.

A set of variables was adjusted for in the fully adjusted models. Educational attainment was categorized according to the International Standard Classification of Education classification (22). Housing tenure was used as a proxy for long-term wealth (dichotomized as owner or non-owner). Individuals were further distinguished between those residing in an urban agglomeration or not (23), and according to whether they had a partner living in the same household. Migration background (Belgian or foreign nationality of origin) was also included as an adjustment variable. Besides socio-economic characteristics, we also accounted for work-related factors: the broader economic sector of the main job, total number of weekly working hours (in main and side job) in categories (24), type of work schedule in the main job (including shift work), and multiple job-holding. The effects of work schedules and working hours on health and mortality have been analyzed in their own right (24–26), and adjusting for these enables us to establish if they act as confounders with regards to the relation under study.

All-cause mortality and mortality from the following underlying major causes was considered: diseases of the circulatory system (ICD10 I00–I99), cancer (ICD10 C00–D48), and all external causes (ICD10 V01–Y98). These major groups of causes were often investigated in previous unemployment- and work-related mortality research (14, 16, 18, 19). As external causes were previously pointed out as a source of excess mortality among non-permanent employees (19), we also specified for the following external sub-causes: transport accidents (ICD10 V01–V99), suicide (ICD10 X60–X84), and falls (ICD10 W00–W19). For approximately 1.5% of the events (around 1.8% and 1.2% for events among men and among women, respectively), no underlying cause of death was known.

### Statistical analyses

Cox proportional hazards models (27, 28) were fitted to analyze the association between non-standard employment and mortality, with age as the underlying timescale, additionally adjusting for age in 5-year categories at the beginning of the follow-up (29). The proportional hazards assumption was assessed using Schoenfeld residuals, and by plotting Kaplan-Meier curves against predicted survival curves (28, 30). Separate models were fitted by gender.

### Sensitivity analysis

To further account for potential confounders, we conducted a propensity score matching (15, 31–34) as part of a sensitivity analysis. This entailed a set of binary matches (34), separately for men and women. These analyses were conducted on a wider subpopulation, which included those with poorer health and a long-standing condition. Scores were calculated and observations outside of the region of common support – individuals with a higher score than the maximum or a lower score than the minimum observed in the other group – were not included in the matched samples (33). Indicators used to calculate the propensity scores included self-rated health and the presence of a disability, as well as all adjustment variables detailed above except the type of work schedule, as this was unlikely to influence take-up of a certain form of employment, being more intrinsically linked to a job. It is strongly recommended to use pre-treatment/pre-exposure variables for establishing the matched sample, however, some of the indicators (such as health) included in the matching could have already been impacted by exposure to certain forms of employment, likely to impact (and introduce bias to) our estimates (32). As a last step, we ran the Cox models (adjusting for all variables used for matching as well as work schedule) on the matched samples. It needs to be noted that as binary matches were conducted, the matched sample of permanent employees (the “control group”) was likely to slightly differ in each case (34). The analyses were carried out using STATA 14.2 (StataCorp, College Station, TX, USA.) and R (35), using the MatchIt package in the latter (36).

## Results

### Description of the research population

As table 1 shows, permanent employees formed the biggest group and fixed-term workers made up the largest non-standard group among both genders. Whereas around a third of male permanent employees and almost half of female permanent employees had tertiary qualifications, this proportion was only 12–13% among seasonal workers and around 19% among casual workers. The levels of educational attainment among female fixed-term workers were nearly comparable to those undertaking permanent employment. The prevalence of migration background was higher among all non-standard than permanent workers. Inequalities in housing tenure were also observed.

**Table 1:** Socio-economic characteristics by employment form.

	N	Education			Migration background (%)	Housing tenure (% owner)
	
		(Pre)primary (%)	Secondary (%)	Tertiary (%)		
Men						
Permanent employment	777 070	7.7	57.5	34.9	12.1	80.9
Non-standard employment forms						
Temporary agency work	6688	14.3	67.1	18.6	34.7	51.7
Seasonal work	658	21.0	66.0	13.1	28.0	57.8
Fixed-term employment	19 709	10.4	47.4	42.3	27.9	63.2
Employment program	5991	14.8	62.5	22.7	16.9	62.9
Casual work or other	865	20.6	59.8	19.7	23.5	65.0
Total	810 981	7.9	57.4	34.8	12.8	80.1
Women						
Permanent employment	579 129	5.1	50.2	44.8	9.9	80.8
Non-standard employment forms						
Temporary agency work	7894	9.2	60.7	30.2	21.2	64.3
Seasonal work	1024	18.9	69.2	11.9	24.1	67.1
Fixed-term employment	34 014	7.2	50.1	42.7	17.1	71.3
Employment program	18 745	8.2	70.6	21.2	10.1	73.8
Casual work or other	2246	12.3	68.8	18.9	15.8	79.8
Total	643 052	5.4	51.0	43.6	10.5	79.9

### Analysis of mortality risks

Table 2 presents the results for all-cause mortality, whereas the cause-specific results are displayed in tables 3 and 4 for men and women, respectively. In total, over 40 000 deaths occurred over the 2001–2014 period. The age-adjusted models revealed that among men, all but casual workers were predisposed to higher risk of all-cause mortality than were permanent workers. The same held for cancer mortality. Holding a temporary agency or a fixed-term job or being employed in an employment program was a predictor for a raised risk of all-cause mortality among women.

**Table 2:** All-cause mortality 2001–2014 by employment form in 2001. Reference category: permanent employment. [HR=hazard ratio; CI=confidence interval from Cox proportional hazards regressions]

Employment type	N	Person-years	Number of deaths 2001–2014	Age-adjusted HR (95% CI)	Fully adjusted ^a^ HR (95% CI)
Men					
Permanent employment	777 070	10 015 194	27 627	1.00	1.00
Non-standard employment forms					
Temporary agency work	6688	84 069	306	1.79 (1.60–2.00)	1.51 (1.35–1.69)
Seasonal work	658	8116	40	1.99 (1.46–2.72)	1.47 (1.07–2.01)
Fixed-term employment	19 709	245 508	735	1.35 (1.26–1.46)	1.26 (1.17–1.35)
Employment program	5991	76 871	332	1.69 (1.52–1.88)	1.29 (1.16–1.44)
Casual work	865	10 810	32	1.10 (0.78–1.56)	0.90 (0.64–1.28)
Women					
Permanent employment	579 129	7 557 646	9833	1.00	1.00
Non-standard employment forms					
Temporary agency work	7894	101 972	153	1.38 (1.18–1.62)	1.28 (1.09–1.51)
Seasonal work	1024	13 186	<20	0.92 (0.57–1.49)	0.81 (0.50–1.31)
Fixed-term employment	34 014	440 248	556	1.18 (1.08–1.28)	1.14 (1.04–1.24)
Employment program	18 745	245 733	342	1.18 (1.06–1.31)	1.06 (0.95–1.19)
Casual work	2246	29 096	50	1.15 (0.87–1.52)	1.15 (0.87–1.52)

a Adjusted for age, educational attainment, living in urban agglomeration, partner in household, migration background, economic sector, housing tenure, weekly working hours, work schedule and multiple job-holding.

**Table 3:** Associations between employment form in 2001 and cause-specific mortality 2001–2014 among men. Hazard ratios (HR) [and 95% confidence intervals (CI)] from Cox proportional hazards regressions.

Cause of death	Permanent employment	Non-standard employment forms				

		Temporary agency work	Seasonal work	Fixed-term employment	Employment program	Casual work or other
				
		HR (95% CI)	HR (95% CI)	HR (95% CI)	HR (95% CI)	HR (95% CI)
Circulatory diseases (N=5791)					
Age-adjusted	1.00	1.89 (1.47–2.44)	2.04 (1.02–4.08)	1.18 (0.99–1.41)	1.77 (1.40–2.25)	1.03 (0.46–2.30)
Fully adjusted ^a^	1.00	1.52 (1.18–1.96)	1.42 (0.71–2.85)	1.07 (0.89–1.28)	1.29 (1.01–1.64)	0.81 (0.36–1.80)
Cancer (N=12 075)						
Age-adjusted	1.00	1.55 (1.27–1.89)	2.10 (1.30–3.37)	1.28 (1.13–1.44)	1.45 (1.20–1.74)	1.08 (0.63–1.86)
Fully adjusted ^a^	1.00	1.38 (1.13–1.68)	1.64 (1.02–2.65)	1.22 (1.08–1.38)	1.21 (1.00–1.46)	0.93 (0.54–1.61)
All external causes (N=4534)						
Age-adjusted	1.00	2.36 (1.90–2.92)	2.30 (1.15–4.61)	1.13 (0.94–1.36)	1.48 (1.12–1.96)	1.08 (0.45–2.60)
Fully adjusted ^a^	1.00	2.07 (1.66–2.56)	1.74 (0.87–3.50)	1.10 (0.91–1.32)	1.12 (0.84–1.49)	0.88 (0.36–2.12)
Transport accidents (N=1036)						
Age-adjusted	1.00	2.10 (1.33–3.30)	6.03 (2.51–14.53)	1.01 (0.68–1.48)	1.31 (0.70–2.45)	N/A ^b^
Fully adjusted ^a^	1.00	1.85 (1.17–2.93)	5.04 (2.07–12.27)	1.04 (0.70–1.55)	1.15 (0.61–2.16)	N/A ^b^
Suicide (N=2378)						
Age-adjusted	1.00	2.34 (1.74–3.13)	1.09 (0.27–4.35)	1.14 (0.89–1.47)	1.48 (1.01–2.18)	1.23 (0.40–3.82)
Fully adjusted ^a^	1.00	2.17 (1.61–2.91)	0.83 (0.21–3.32)	1.15 (0.90–1.48)	1.12 (0.76–1.66)	1.02 (0.33–3.18)
Fall (N=359)						
Age-adjusted	1.00	2.70 (1.20–6.06)	4.05 (0.57–28.82)	1.59 (0.87–2.90)	0.40 (0.06–2.81)	N/A ^b^
Fully adjusted ^a^	1.00	2.34 (1.03–5.29)	3.49 (0.48–25.29)	1.41 (0.77–2.60)	0.28 (0.04–2.01)	N/A ^b^

a Adjusted for age, educational attainment, living in urban agglomeration, partner in household, migration background, economic sector, housing tenure, weekly working hours, work schedule and multiple job-holding.

b No point estimate was calculated due to lack of events.

**Table 4:** Associations between employment form in 2001 and cause-specific mortality 2001–2014 among women. Hazard ratios (HR) [and 95% confidence intervals (CI)] from Cox proportional hazards regressions.

Cause of death	Permanent employment	Non-standard employment forms				

		Temporary agency work	Seasonal work	Fixed-term employment	Employment program	Casual work or other
				
		HR (95% CI)	HR (95% CI)	HR (95% CI)	HR (95% CI)	HR (95% CI)
Circulatory diseases (N=1300)						
Age-adjusted	1.00	1.80 (1.19–2.72)	1.40 (0.45–4.34)	1.25 (0.98–1.60)	1.53 (1.16–2.02)	1.38 (0.66–2.91)
Fully adjusted ^a^	1.00	1.57 (1.03–2.38)	1.06 (0.34–3.32)	1.17 (0.91–1.50)	1.28 (0.96–1.71)	1.34 (0.63–2.84)
Cancer (N=6484)						
Age-adjusted	1.00	1.05 (0.83–1.34)	0.54 (0.24–1.20)	1.05 (0.93–1.18)	1.22 (1.06–1.40)	0.99 (0.67–1.45)
Fully adjusted ^a^	1.00	0.99 (0.78–1.26)	0.50 (0.22–1.12)	1.04 (0.92–1.17)	1.15 (1.00–1.33)	0.97 (0.66–1.43)
All external causes (N=1158)						
Age-adjusted	1.00	2.02 (1.38–2.96)	2.23 (0.83–5.95)	1.15 (0.90–1.48)	0.89 (0.62–1.28)	0.99 (0.37–2.64)
Fully adjusted ^a^	1.00	1.92 (1.30–2.83)	1.91 (0.71–5.17)	1.05 (0.82–1.35)	0.73 (0.51–1.07)	1.00 (0.37–2.67)
Transport accidents (N=227)						
Age-adjusted	1.00	2.91 (1.43–5.90)	N/A ^b^	1.01 (0.56–1.81)	1.07 (0.50–2.27)	1.34 (0.19–9.55)
Fully adjusted ^a^	1.00	2.52 (1.22–5.17)	N/A ^b^	0.87 (0.48–1.58)	0.80 (0.37–1.74)	1.18 (0.16–8.59)
Suicide (N=619)						
Age-adjusted	1.00	1.52 (0.84–2.76)	1.04 (0.15–7.42)	1.10 (0.78–1.55)	0.98 (0.61–1.56)	0.92 (0.23–3.69)
Fully adjusted ^a^	1.00	1.45 (0.79–2.65)	0.99 (0.14–7.11)	1.02 (0.72–1.45)	0.82 (0.50–1.32)	0.96 (0.24–3.87)
Fall (N=90)						
Age-adjusted	1.00	2.12 (0.52–8.63)	N/A ^b^	1.00 (0.37–2.74)	0.39 (0.05–2.81)	N/A ^b^
Fully adjusted ^a^	1.00	2.05 (0.49–8.48)	N/A ^b^	0.83 (0.30–2.29)	0.27 (0.04–1.99)	N/A ^b^

a Adjusted for age, educational attainment, living in urban agglomeration, partner in household, migration background, economic sector, housing tenure, weekly working hours, work schedule and multiple job-holding.

b No point estimate was calculated due to lack of events.

Further adjustment shows that some of the associations can be explained by permanent and non-standard workers’ differing socio-demographic and work-related characteristics. Among men, adjusting for educational attainment and housing tenure, and among women, accounting for the economic sector of main job and housing tenure reduced the point estimates to the largest extent. Additional adjustment for total weekly working hours, work schedule and multiple job-holding, however, did not attenuate the estimates of employment forms much (data not shown). As shown in the supplementary material (www.sjweh.fi/show_abstract.php?abstract_id=3931), tables S1 and S2, among men, working >40 hours a week, whereas among women, working less than the conventional full-time hours was linked to a reduced risk of all-cause mortality. Working shifts was linked to a higher risk of mortality from all causes, as well as from external causes and suicide among men, compared to working sliding hours.

Among men after adjustment, temporary agency, seasonal and fixed-term workers as well as those in employment programs in 2001 experienced excess risk of all-cause mortality in the subsequent 13 years compared to their permanently employed counterparts. We found an over twofold increased hazard ratio (HR) for all external causes [HR 2.07, 95% confidence interval (CI) 1.66–2.56], suicide (HR 2.17, 95% CI 1.61–2.91) and fall (HR 2.34, 95% CI 1.03–5.29) among male temporary agency workers, and an over five times higher risk of death due to transport accidents among male seasonal workers (HR 5.04, 95% CI 2.07–12.27). This is the highest adjusted HR exhibited in the study, although the CI is wide due to the small number of events. Male temporary agency (HR 1.38, 95% CI 1.13–1.68), seasonal (HR 1.64, 95% CI 1.02–2.65), fixed-term workers (HR 1.22, 95% CI 1.08–1.38) and those in employment programs (HR 1.21, 95% CI 1.00–1.46) experienced raised adjusted cancer mortality in comparison to permanent employees, and those employed within the framework of an employment program or holding a temporary agency contract were at heightened risk of death from circulatory diseases.

Among women, holding a temporary agency job, compared to being permanently employed, was a predictor for a higher risk of mortality from all causes (HR 1.28, 95% CI 1.09–1.51), circulatory diseases (HR 1.57, 95% CI 1.03–2.38), all external causes (HR 1.92, 95% CI 1.30–2.83) and transport accidents (HR 2.52, 95% CI 1.22–5.17) after adjustment. All-cause mortality was also slightly increased among women undertaking fixed-term jobs (HR 1.14, 95% CI 1.04–1.24), but a break-down by causes did not show elevated point estimates.

### Sensitivity analysis

Additional analyses on the matched samples (as presented in supplementary table S3) showed that for male seasonal workers, the association with all-cause and cancer mortality was no longer present in the matched sample. This, however, cannot be said about male temporary agency workers, where the HR of their employment situation remained to be elevated for all causes investigated, apart from falls, after adjustment. The slightly elevated all-cause mortality found for male fixed-term employees and men in employment programs was also not explained by known confounding factors. Among women, fixed-term employees had a slightly increased relative risk of all-cause mortality in comparison to permanent employees.

## Discussion

Our study, which to our knowledge is the first one to assess associations between forms of non-standard employment and mortality using population-wide data, revealed considerable mortality inequalities within the salaried employee population in Belgium. Over the subsequent 13 years and three months of follow-up, certain non-standard workers were at increased risk of death compared to permanently employed workers. Our analyses add to existing evidence on the negative relationship between non-standard and temporary employment and health (6–9), and in particular to the handful of studies which looked at mortality outcomes (17–19) in the past. The considerable mortality inequalities we found indicate that exposure to certain forms of employment that deviate from permanent employment – however brief that may be – could be associated with a ‘health scar’ (11, 12). More broadly speaking, our investigation also links to unfolding discussions and growing evidence on the health implications of precarious employment (37).

An important contribution of this study lies in showing that the different forms of non-standard employment under examination were associated with different mortality risks and patterns. Particularly male temporary agency and seasonal workers were predisposed to elevated mortality relative to their permanent counterparts. This points to the heterogeneity between types of non-permanent workers when it comes to health and mortality, as observed – albeit differently – in prior research (17). Our results are also somewhat in line with a previous study, which demonstrated a health disadvantage for a group of non-permanent employees comprising seasonal and temporary agency workers compared to permanent workers – albeit not for fixed-term workers (21).

In the case of men in seasonal employment, however, accounting for socio-demographic and work-related characteristics often attenuated their mortality risks. Additional analyses on matched samples also underlined the role of confounding for this group of workers. Male temporary agency workers’ relative mortality risks, on the other hand, remained quite consistently raised after full adjustment, as well as matching. They demonstrated a substantial mortality disadvantage, despite the legislative framework this form of work is subject to in Belgium (38). Our results overall underline that, from a health perspective, some non-permanent jobs (such as casual employment) might overall be less disadvantageous and leave its holders less vulnerable than others (like temporary agency work) (see 39). This study overall reveals the presence of a core and periphery (18, 21) within the Belgian labor force from a mortality perspective, although it also shows that this distinction may be limiting. Some non-permanent jobs might be more peripheral than others, indicating the need to move beyond a binary conceptualization of labor market segmentation. Temporary agency work, in particular, could cluster together with other adverse aspects of employment, putting workers at the lowest spectrum of employment quality (40), whereas some non-permanent jobs might be more advantageous in this regard. Workers in various work arrangements might also differ in their ability to obtain permanent employment later on, and in their likelihood to have transitioned out of or into unemployment before or after 2001.

A further insight our analysis has provided pertains to the specific causes of death. Excess mortality from external causes in temporary workers has been demonstrated in a prior study (19). Temporary agency workers were, after adjustment, and in comparison to, permanent employees, at an around twofold risk of dying from all external causes. When delving into the sub-causes, we found similarly elevated fall mortality in male temporary agency workers with conventional regression adjustment, although the low number of events needs to be stressed. Workplace accidents resulting from unsafe working conditions, faster working tempo or insufficient job-specific knowledge among this group could be explaining factors in this association (5, 41, 42). Our results are in line with earlier findings (41) which indicated that temporary employment as a whole was particularly strongly associated with fatal work-related injuries (which covered some types of falls). Male temporary agency workers were also at a heightened relative risk of death by suicide. Transport accidents were causes of an excess risk of mortality in temporary agency and male seasonal workers. Further investigation should probe into the underlying mechanisms. Finally, contrary to earlier studies (18, 19), we found an elevated risk of *both* circulatory and cancer mortality (after adjustment) in some groups of non-permanent workers, compared to permanent employees, although some of this mortality disadvantage could be ascribed to confounding factors. Our data’s population-wide coverage, and the possibility of differentiating between various sub-groups of non-standard workers probably revealed some previously hidden mortality inequalities in this regard.

Our study also showcased strong gender differences. Female non-standard workers exhibited smaller HR overall than did their male counterparts and demonstrated less pronounced mortality patterns. This goes contrary to arguments that precarious employment could be more detrimental to women’s than men’s health (43), although our findings might be pertinent to the specific health outcome under study (44). There are indications that non-standard employment as a whole (including part-time employment) has a gendered distribution among households in Belgium (45). Female non-standard workers’ unstable or low incomes could thus often be supplemented by their partner’s (potentially more stable) wages, reflected in female temporary employee’s lowers odds of being below the poverty line compared to their male counterparts (46). Overall, the less prominent position of employment in many women’s lives may attenuate the mortality outcomes found in this study. Future research should strive to gain a broader household-perspective (45) when analyzing the health and mortality implications of non-standard employment.

Some limitations of our study need to be mentioned. Although the census provided information on all individuals in our subpopulation, it did so at one point in time. Individuals’ broader employment trajectories, including the length of exposure to unemployment, which has been linked to subsequent mortality (14–16), could not be accounted for, nor did we have information on the length of time individuals spent in specific non-standard employment arrangements. There was also no information available in the census on individuals’ lifestyle (eg, alcohol consumption or smoking) (18, 19), nor could we account for health status and other aspects prior to the census, a limitation for our sensitivity analyses. Restricting the analyses to healthy individuals with no long-standing illness or disability at the time of the census – and accounting for health status as part of our matching – meant however that we were able to reduce some of the potential health selection effects. Lastly, we could not fully construct a multidimensional index of precarious employment, and instead mostly relied on contractual instability, which, albeit widely used as a measure (47), has been shown to constitute only a partial proxy of employment precariousness (48). Nevertheless, we did additionally account for working hours, work schedule and multiple job-holding in our analyses.

Overall, our study’s main strength stems from the register-based data we were able to draw on. The population-wide coverage and 13 years of mortality follow-up allowed us to evaluate cause-specific mortality, differentiate between various sub-groups and study long-term outcomes. This unique dataset enabled us to demonstrate considerable relative excess mortality for some groups of non-standard employees compared to permanent workers. Moreover, due to the large sample size, we were able to study mortality for employment forms separately for women and men, revealing substantial differences in patterns and extents of risks. In conclusion, we showed that taking non-permanent workers as one group conceals mortality inequalities within them. A dichotomous core-periphery understanding, therefore, might be less helpful in explaining mortality inequalities within the workforce. Further research on work-related health should therefore strive to study groups of workers in various contractual arrangements separately, while also accounting for spells of unemployment and changes between jobs with differing levels of precarity. This prerequisites access to adequate longitudinal data with information on trajectories and different aspects of working conditions. We also highlighted the need for studying the long-term health effects of employment experiences, particularly among non-permanent workers. Monitoring the latter’s health can be challenging from an occupational health and safety perspective (49), but is certainly warranted. All in all, policy-makers should pay more attention to non-standard employment as a potential work-related health determinant.

### Concluding remarks

Our investigation shows that male workers in temporary agency contracts in 2001 in particular exhibited relative excess mortality compared to permanent workers during a more than 13-year follow-up period and that external causes of death played an important role in this association. This association persisted after accounting for differing characteristics between temporary agency and permanent workers.

### Conflict of interest

The authors declare no conflicts of interest.

## Supplementary material

Supplementary material
