# Supplementary material for: Non-standard employment and mortality in Belgian workers: A census-based investigation
Source: Scand J Work Environ Health. 2021 Mar 1;47(2):108–16. doi: 10.5271/sjweh.3931 (PMC8114567; doi:10.5271/sjweh.3931)
Supplement: Supplementary material [file SJWEH-47-108-S001.pdf]

# Non-standard employment and mortality in Belgian workers: A census-based investigation[1]

by Rebeka Balogh, MSc,[2] Sylvie Gadeyne, PhD, Christophe Vanroelen, PhD

1. *Supplementary tables*
2. *Correspondence to: Rebeka Balogh, Department of Sociology, Vrije Universiteit Brussel, Pleinlaan 2, 1050 Brussels, Belgium. [E-mail: Rebeka.Balog*h@vub.be]

Table S1 - Associations between weekly working hours and work schedule in 2001 and all-cause and cause-specific mortality 2001-2014 among men after full adjustment. Hazard ratios (and 95% confidence intervals) from Cox proportional hazards regressions

| | All-cause (n= 29 072) | Circulatory diseases (n=5791) | Cancer (n=12 075) | All external causes (n=4534) | Transport accidents (n=1036) | Suicide (n=2378) | Fall (n=359) |
|---|---|---|---|---|---|---|---|
| **Work schedule** | | | | | | | |
| Staggered (sliding hours) (N=128 949) | 1.00 | 1.00 | 1.00 | 1.00 | 1.00 | 1.00 | 1.00 |
| Fixed (N=471 242) | 1.07 (1.03-1.10) | 1.03 (0.95-1.11) | 1.09 (1.03-1.15) | 1.08 (0.98-1.19) | 1.13 (0.92-1.39) | 1.14 (0.99-1.30) | 0.81 (0.60-1.09) |
| Interrupted (N=25 814) | 1.09 (1.01-1.17) | 1.22 (1.04-1.42) | 1.06 (0.94-1.19) | 1.08 (0.90-1.30) | 0.98 (0.66-1.45) | 1.14 (0.88-1.46) | 0.97 (0.52-1.83) |
| Flexible (set by the employer) (N=112 102) | 1.09 (1.04-1.14) | 1.01 (0.92-1.12) | 1.06 (0.99-1.14) | 1.20 (1.07-1.35) | 1.18 (0.93-1.51) | 1.31 (1.12-1.53) | 0.73 (0.49-1.11) |
| Shift work (N= 70 403) | 1.08 (1.02-1.14) | 0.92 (0.81-1.04) | 1.07 (0.98-1.16) | 1.21 (1.06-1.38) | 1.09 (0.82-1.45) | 1.32 (1.11-1.59) | 1.01 (0.64-1.58) |
| Other (N=2471) | 1.07 (0.86-1.33) | 1.03 (0.63-1.69) | 0.82 (0.55-1.21) | 1.02 (0.57-1.80) | 0.75 (0.19-3.03) | 1.17 (0.55-2.48) | 0.88 (0.12-6.35) |

| | | | | | | | |
|---|---|---|---|---|---|---|---|
| **Working hours** | | | | | | | |
| More than 48 (N= 109 862) | 0.95 (0.91-0.98) | 0.93 (0.85-1.02) | 0.98 (0.92-1.05) | 0.85 (0.76-0.95) | 0.99 (0.80,1.23) | 0.83 (0.71-0.96) | 0.74 (0.49-1.10) |
| 41 to 48 (N=90 293) | 0.89 (0.86-0.93) | 0.84 (0.76-0.93) | 0.98 (0.92-1.05) | 0.82 (0.73-0.92) | 0.80 (0.63,1.03) | 0.83 (0.71-0.98) | 1.08 (0.74-1.56) |
| 35 to 40 (N=568 250) | 1.00 | 1.00 | 1.00 | 1.00 | 1.00 | 1.00 | 1.00 |
| 20 to 34 (N=32 264) | 1.00 (0.94-1.06) | 0.99 (0.87-1.12) | 0.98 (0.89-1.07) | 1.08 (0.93-1.25) | 0.91 (0.65-1.28) | 1.17 (0.96-1.42) | 1.22 (0.74-1.98) |
| Less than 20 (N=10 312) | 0.98 (0.90-1.08) | 1.07 (0.89-1.29) | 0.90 (0.78-1.03) | 1.03 (0.81-1.32) | 1.01 (0.59-1.72) | 1.01 (0.72-1.44) | 1.09 (0.48-2.48) |

adjusted for employment form, age, educational attainment, living in urban agglomeration, partner in household, migration background, economic sector, housing tenure and multiple job-holding.

N/A: no point estimate was calculated due to lack of events

Table S2 - Associations between total working hours and work schedule in 2001 and all-cause and cause-specific mortality 2001-2014 among women after full adjustment. Hazard ratios (and 95% confidence intervals) from Cox proportional hazards regressions.

| | All-cause (n= 10 951) | Circulatory diseases (n=1300) | Cancer (n=6484) | All external causes (n=1158) | Transport accidents (n=227) | Suicide (n=619) | Fall (n=90) |
|---|---|---|---|---|---|---|---|
| **Work schedule** | | | | | | | |
| Staggered (sliding hours) (N=106 968) | 1.00 | 1.00 | 1.00 | 1.00 | 1.00 | 1.00 | 1.00 |
| Fixed (N = 409 885) | 0.99 (0.94-1.05) | 1.03 (0.88-1.21) | 0.98 (0.92-1.05) | 0.93 (0.79-1.10) | 0.79 (0.55-1.15) | 1.09 (0.86-1.39) | 0.78 (0.45-1.36) |
| Interrupted (N=27 783) | 1.00 (0.90-1.11) | 0.97 (0.72-1.31) | 0.91 (0.79-1.04) | 1.30 (0.98-1.72) | 1.31 (0.72-2.38) | 1.48 (1.00-2.18) | 0.41 (0.09-1.80) |
| Flexible (set by the employer) (N=72 517) | 0.96 (0.89-1.04) | 0.87 (0.70-1.10) | 0.96 (0.87-1.06) | 1.02 (0.81-1.27) | 1.04 (0.63-1.70) | 1.15 (0.84-1.57) | 0.88 (0.39-1.99) |
| Shift work (24 344) | 1.01 (0.90-1.13) | 1.11 (0.80-1.53) | 0.88 (0.75-1.03) | 1.29 (0.95-1.74) | 0.82 (0.39-1.72) | 1.62 (1.09-2.41) | 0.92 (0.26-3.23) |
| Other (N=1555) | 0.80 (0.51-1.25) | 1.58 (0.59-4.27) | 0.56 (0.28-1.12) | 2.46 (1.15-5.24) | 3.44 (0.83-14.35) | 3.55 (1.44-8.78) | N/A |
| **Working hours** | | | | | | | |
| More than 48 (N=26 627) | 0.90 (0.81-1.00) | 0.97 (0.71-1.31) | 0.91 (0.79-1.04) | 0.82 (0.60-1.14) | 0.80 (0.38-1.67) | 0.75 (0.47-1.21) | 0.23 (0.03-1.67) |
| 41 to 48 (35 264) | 0.96 (0.88-1.05) | 0.90 (0.68-1.20) | 1.03 (0.91-1.15) | 0.73 (0.54-0.99) | 0.70 (0.35-1.40) | 0.82 (0.55-1.23) | 0.35 (0.08-1.49) |
| 35 to 40 (303 399) | 1.00 | 1.00 | 1.00 | 1.00 | 1.00 | 1.00 | 1.00 |
| 20 to 34 (N=199 046) | 0.90 (0.86-0.94) | 0.86 (0.75-0.98) | 0.91 (0.85-0.96) | 0.90 (0.78-1.03) | 0.80 (0.58-1.10) | 0.98 (0.81-1.18) | 0.86 (0.52-1.41) |

| | | | | | | | |
|---|---|---|---|---|---|---|---|
| Less than 20<br>(78 716) | 0.91<br>(0.85-0.96) | 0.85<br>(0.72-1.02) | 0.90<br>(0.83-0.98) | 0.98<br>(0.82-1.19) | 0.91<br>(0.59-1.39) | 1.20<br>(0.94-1.53) | 0.86<br>(0.44-1.70) |

adjusted for employment form, age, educational attainment, living in urban agglomeration, partner in household, migration background, economic sector, housing tenure and multiple job-holding.

N/A: no point estimate was calculated due to lack of events

Table S3 - Type of employment in 2001 and all-cause and cause-specific mortality 2001-2014 on matched samples (binary matching) after full adjustment. Hazard ratios (and 95% confidence intervals) from Cox proportional hazards regressions

| MEN | | | | | | | |
|---|---|---|---|---|---|---|---|
| | All-cause | Circulatory diseases | Cancer | All external causes | Transport accidents | Suicide | Fall |
| Permanent empl. (N= 970 775) | 1.00 | 1.00 | 1.00 | 1.00 | 1.00 | 1.00 | 1.00 |
| Temporary agency work (N= 9064) | 1.41 (1.29-1.54) | 1.46 (1.20-1.77) | 1.31 (1.12-1.53) | 1.85 (1.54-2.22) | 1.90 (1.30-2.78) | 1.70 (1.31-2.20) | 1.81 (0.89-3.66) |
| *N* | *979 839* | *979 839* | *979 839* | *979 839* | *979 839* | *979 839* | *979 839* |
| Permanent empl. (N= 950 104) | 1.00 | 1.00 | 1.00 | 1.00 | 1.00 | 1.00 | 1.00 |
| Seasonal work (N=947) | 1.16 (0.90-1.49) | 1.26 (0.74-2.13) | 1.16 (0.77-1.75) | 1.56 (0.88-2.75) | 3.36 (1.38-8.16) | 1.20 (0.50-2.89) | 3.59 (0.88-14.63) |
| *N* | *951 051* | *951 051* | *951 051* | *951 051* | *951 051* | *951 051* | *951 051* |
| Permanent empl. (N=972 774) | 1.00 | 1.00 | 1.00 | 1.00 | 1.00 | 1.00 | 1.00 |
| Fixed-term work (N= 25 748) | 1.22 (1.15-1.29) | 1.06 (0.92-1.21) | 1.21 (1.10-1.33) | 1.17 (1.02-1.36) | 1.14 (0.83-1.56) | 1.19 (0.98-1.44) | 1.24 (0.75-2.06) |
| *N* | *998 522* | *998 522* | *998 522* | *998 522* | *998 522* | *998 522* | *998 522* |
| Permanent empl. (N= 969 215) | 1.00 | 1.00 | 1.00 | 1.00 | 1.00 | 1.00 | 1.00 |
| Empl. program (N=9017) | 1.22 (1.12-1.32) | 1.19 (1.00-1.42) | 1.18 (1.03-1.35) | 1.22 (0.99-1.50) | 1.50 (0.98-2.32) | 1.09 (0.81-1.46) | 0.84 (0.37-1.91) |
| *N* | *978 232* | *978 232* | *978 232* | *978 232* | *978 232* | *978 232* | *978 232* |
| Permanent empl. (N=964 861) | 1.00 | 1.00 | 1.00 | 1.00 | 1.00 | 1.00 | 1.00 |
| Casual work or other (N=1291) | 0.99 (0.79-1.24) | 0.86 (0.51-1.46) | 0.87 (0.59-1.29) | 0.99 (0.53-1.85) | N/A | 1.10 (0.49-2.47) | 1.13 (0.16-8.12) |
| *N* | *966 152* | *966 152* | *966 152* | *966 152* | *966 152* | *966 152* | *966 152* |

| WOMEN | | | | | | | |
|---|---|---|---|---|---|---|---|
| | All-cause | Circulatory diseases | Cancer | All external causes | Transport accidents | Suicide | Fall |
| Permanent empl. (N=693 666) | 1.00 | 1.00 | 1.00 | 1.00 | 1.00 | 1.00 | 1.00 |
| Temporary agency work (N=9691) | 1.14 (0.99-1.30) | 1.51 (1.07-2.13) | 0.91 (0.74-1.11) | 1.69 (1.22-2.35) | 2.73 (1.51-4.93) | 1.25 (0.75-2.10) | 1.36 (0.33-5.57) |
| *N* | *703 357* | *703 357* | *703 357* | *703 357* | *703 357* | *703 357* | *703 357* |
| Permanent empl. (N=684 014) | 1.00 | 1.00 | 1.00 | 1.00 | 1.00 | 1.00 | 1.00 |
| Seasonal work (N=1300) | 0.88 (0.61-1.27) | 1.17 (0.48-2.83) | 0.67 (0.38-1.18) | 1.83 (0.81-4.14) | N/A | 1.83 (0.58-5.79) | N/A |
| *N* | *685 314* | *685 314* | *685 314* | *685 314* | *685 314* | *685 314* | *685 314* |
| Permanent empl. (N=694 209) | 1.00 | 1.00 | 1.00 | 1.00 | 1.00 | 1.00 | 1.00 |
| Fixed-term work (N=41 548) | 1.11 (1.04-1.19) | 1.18 (0.97-1.43) | 1.01 (0.92-1.11) | 1.14 (0.93-1.38) | 1.09 (0.68-1.73) | 1.06 (0.80-1.39) | 1.45 (0.75-2.79) |
| *N* | *735 757* | *735 757* | *735 757* | *735 757* | *735 757* | *735 757* | *735 757* |
| Permanent empl. (N= 693 486) | 1.00 | 1.00 | 1.00 | 1.00 | 1.00 | 1.00 | 1.00 |
| Empl. program (N=24 453) | 1.02 (0.94-1.11) | 1.15 (0.91-1.45) | 1.08 (0.97-1.21) | 0.74 (0.55-0.98) | 0.75 (0.38-1.50) | 0.78 (0.53-1.14) | 0.34 (0.08-1.42) |
| *N* | *717 939* | *717 939* | *717 939* | *717 939* | *717 939* | *717 939* | *717 939* |
| Permanent empl. (N= 676 902) | 1.00 | 1.00 | 1.00 | 1.00 | 1.00 | 1.00 | 1.00 |
| Casual work or other (N=2841) | 1.05 (0.84-1.32) | 0.92 (0.46-1.86) | 0.94 (0.68-1.29) | 1.54 (0.82-2.89) | 0.74 (0.10-5.32) | 1.85 (0.82-4.18) | N/A |
| *N* | *679 743* | *679 743* | *679 743* | *679 743* | *679 743* | *679 743* | *679 743* |

Matching was conducted on age at baseline, educational attainment, living in urban agglomeration, partner in household, migration background, economic sector, housing tenure, weekly working hours, multiple job-holding, self-rated health, long-term illness. Fully adjusted model: regression adjusted by all matching variables and work schedule

N/A: no estimate calculated due to lack of events in either of the groups
